# A Novel ENU-Induced *Mfn2* Mutation Causes Motor Deficits in Mice without Causing Peripheral Neuropathy

**DOI:** 10.3390/biology12070953

**Published:** 2023-07-03

**Authors:** Timothy J. Hines, Janice Bailey, Hedi Liu, Anyonya R. Guntur, Kevin L. Seburn, Samia L. Pratt, Jonathan R. Funke, Lisa M. Tarantino, Robert W. Burgess

**Affiliations:** 1 The Jackson Laboratory, Bar Harbor, ME 04609, USA; 2 Department of Genetics, The University of North Carolina, Chapel Hill, NC 27599, USA; 3 Center for Molecular Medicine, Maine Health Institute for Research, Scarborough, ME 04074, USA; 4 Neuroscience Program, Graduate School of Biomedical Sciences, Tufts University, Boston, MA 02111, USA

**Keywords:** Charcot–Marie–Tooth disease, CMT2A, mitofusin 2, neuromuscular disease

## Abstract

**Simple Summary:**

The fission and fusion of mitochondria are important processes for maintaining mitochondrial health. One of the proteins responsible for mediating mitochondrial fusion, mitofusin 2 (MFN2), has over 100 known mutations that cause Charcot–Marie–Tooth disease type 2A (CMT2A). This disease causes the nerves that control your muscles to degenerate, leading to muscle atrophy and weakness, problems walking, and other related symptoms. In this paper, we describe a mouse line with a recessive mutation in the *Mfn2* gene (Leu643Pro) that causes a similar set of symptoms, including abnormal gait, weight loss, and decreased muscular endurance. However, further analysis of these mice revealed signs of skeletal muscle dysfunction (including smaller mitochondria) and bone abnormalities, with little evidence of axon degeneration typical of CMT2A. While this makes these mice a poor model for CMT2A, they are the first reported mouse line with a mutation in the transmembrane domain, a region critical for MFN2′s role in mitochondrial fusion. For this reason, we believe these mice will be a valuable tool for scientists interested in studying the biological functions of MFN2.

**Abstract:**

Mitochondrial fission and fusion are required for maintaining functional mitochondria. The mitofusins (MFN1 and MFN2) are known for their roles in mediating mitochondrial fusion. Recently, MFN2 has been implicated in other important cellular functions, such as mitophagy, mitochondrial motility, and coordinating endoplasmic reticulum-mitochondria communication. In humans, over 100 *MFN2* mutations are associated with a form of inherited peripheral neuropathy, Charcot–Marie–Tooth disease type 2A (CMT2A). Here we describe an ENU-induced mutant mouse line with a recessive neuromuscular phenotype. Behavioral screening showed progressive weight loss and rapid deterioration of motor function beginning at 8 weeks. Mapping and sequencing revealed a missense mutation in exon 18 of *Mfn2* (T1928C; Leu643Pro), within the transmembrane domain. Compared to wild-type and heterozygous littermates, *Mfn2*^L643P/L643P^ mice exhibited diminished rotarod performance and decreases in activity in the open field test, muscular endurance, mean mitochondrial diameter, sensory tests, mitochondrial DNA content, and MFN2 protein levels. However, tests of peripheral nerve physiology and histology were largely normal. Mutant leg bones had reduced cortical bone thickness and bone area fraction. Together, our data indicate that *Mfn2*^L643P^ causes a recessive motor phenotype with mild bone and mitochondrial defects in mice. Lack of apparent nerve pathology notwithstanding, this is the first reported mouse model with a mutation in the transmembrane domain of the protein, which may be valuable for researchers studying MFN2 biology.

## 1. Introduction

Mitochondria are double membrane-bound organelles that participate in countless critical cellular functions, most famously the production of chemical energy in the form of ATP. In addition to producing power, mitochondria form a tubular network throughout the cell, mediating several essential cellular activities, such as maintaining intracellular calcium homeostasis, responding to cell stress, determining cell fate, and executing programmed cell death [1,2,3]. Given their indispensable nature as a hub for so many vital cellular activities, it is important to preserve mitochondrial health.

Mitochondrial dynamics, the processes of mitochondrial transport, fission, and fusion, are critical for the proper maintenance and function of these multi-faceted organelles [4,5]. Mitochondrial fission, mediated by two large GTPases, dynamin-related protein 1 (DRP1) and dynamin 2 (DNM2), produces two or more mitochondria from a single mitochondrion, which can then be trafficked to different areas of the cell to replenish damaged mitochondria or participate in energy-intensive processes, such as axon elongation or synaptic transmission [5,6,7]. Fusion, on the other hand, involves the merging of two or more mitochondria into one larger mitochondrion, which can restore mitochondrial health through the complementation of their contents including mitochondrial genomes. This process requires separate fusion events for the inner and outer mitochondrial membranes (IMM and OMM) mediated by large GTPases, optic atrophy 1 (OPA1) and the mitofusins (MFN1 and MFN2), respectively [5,8,9,10]. Fission and fusion are in constant flux adjusting mitochondrial size, shape, and distribution for the rapidly evolving demands of cells all over the body. Therefore, when these processes are out of balance, mitochondria are not as able to efficiently cater to the needs of the cell or tissue, resulting in disease [4,5]. Furthermore, fusion is critical for maintaining mitochondrial DNA (mtDNA) integrity and alterations in mtDNA content have been associated with myriad diseases including Alzheimer’s and Parkinson’s Disease, multiple types of myopathy, and type II diabetes. Accordingly, mtDNA content is used as a marker for mitochondrial health [11,12].

Mammalian mitofusins consist of an N-terminal GTPase domain, one nearby coiled-coil domain called a heptad repeat 1 (HR1) and another (HR2) at the C-terminus, and one transmembrane domain (TM) between HR1 and HR2, which orients the protein so that the C-terminus (containing HR2 and redox-sensitive cysteine residues) is within the intermembrane space (IMS) and the N-terminal GTPase domain resides in the cytoplasm. MFN2 also has a proline-rich region between the HR1 and TM domains, which is absent in MFN1 [13]. Oxidation of the cysteine residues in the IMS leads to disulfide bond formation between mitofusin molecules within the same OMM causing oligomerization, while the N-terminal GTPase coordinates mitochondrial tethering with mitofusins on other mitochondria [13]. Therefore, maintaining the integrity of the transmembrane domain is requisite for preserving both the redox-sensitive oligomerization of mitofusin molecules in *cis* and the GTPase domain-mediated interaction of mitofusin molecules in *trans*.

Charcot–Marie–Tooth disease (CMT) is a rare neuromuscular disease that generally affects the motor and sensory neurons with the longest, largest caliber axons. In addition to their high energy demand, these neurons are faced with the unique challenge of maintaining mitochondria along the full length of their axons, which in humans can extend up to 1 m from the cell body and nucleus to a nerve terminal. This may explain why mutations in many components of the mitochondrial fusion/fission machinery are disproportionately linked to neurodegenerative diseases [14,15,16]. For example, over 100 mutations in *MFN2* have been linked to Charcot–Marie–Tooth disease type 2A (CMT2A) [17,18,19]. Approximately 90% of CMT2A cases are inherited in an autosomal dominant manner, while 10% are recessive [20]. CMT2A is phenotypically heterogeneous with age of onset being the most predictive marker of disease severity. Patients who present with symptoms earlier tend to have more severe sensorimotor neuropathy [21,22,23,24,25,26]. Interestingly, no known *MFN1* mutations cause peripheral neuropathy, which may be due to the relatively low expression of MFN1 in neurons [27,28]. This, amongst a large body of other work, suggests that the mitofusins have distinct functions in addition to their complementary role in fusion [27,28,29,30,31,32]. Indeed, MFN1, but not MFN2, is sufficient to mediate outer membrane fusion with OPA1, while MFN2 has been uniquely implicated in the formation of mitochondria-ER contact sites (MERCS), regulation of long-distance mitochondrial transport in neurons, and modulating differentiation of multiple cell types in bone [5,17,19,31,33,34,35,36]. The biochemical details of how MFN2 carries out these numerous diverse functions are actively being researched using the most recent sequencing, imaging, and structural biology technologies.

Another powerful set of tools in the mitochondria researchers’ toolbox are the numerous cell and animal models with different MFN2 perturbations. However, one ongoing problem in disease-related MFN2 research is the lack of a knock-in mouse that accurately models the genetics AND the severe, early-onset peripheral neuropathy phenotypes commonly seen in patients with CMT2A, despite a great deal of effort to produce such mouse models [27,37,38,39]. Patients often have early and severe disease presentation, with many being wheelchair-bound by their teenage years [38]. However, mice with dominant patient mutations knocked into their endogenous *Mfn2* gene locus to more accurately mimic the disease genetics have not produced the expected neuropathy phenotype as heterozygotes, and homozygotes died embryonically or before weaning [27,39]. Other available models that recapitulate some aspects of the disease, like peripheral axon degeneration, use a transgenic approach, which can create complications such as tissue-specific transgene expression, and overexpression artifacts, making any attempted gene therapy trials difficult to interpret [28,37,40,41,42]. Regardless, these models are still extremely valuable and illustrate specific functions of the gene/protein or new ways that well-known processes, such as mitochondrial dynamics, can go wrong to cause disease.

*N*-Ethyl-*N*-nitrosourea (ENU) is a mutagen commonly used to generate mice with novel phenotypes, including those resembling human disease symptoms. The mapping and sequencing of the genomes of these mice can be used to identify the genetic mechanisms underlying the observed disease phenotype [43]. This method, known as a forward, or phenotype-based genetic screen is useful for identifying currently unknown disease-causing genes and further resolving the cellular pathways involved. An ENU-induced mutagenesis screen conducted at the Genomics Institute of the Novartis Research Foundation (GNF) included several high throughput phenotyping pipelines to measure neurobehavioral, neurosensory, metabolic, immune, and inflammation parameters and produced a mouse line with potential signs of a recessively inherited neuromuscular disease. Genetic mapping and sequencing identified a mutation in exon 18 of *Mfn2* (T1928C; L643P), which resides in the highly conserved TM domain [13,44].

To date (March 2023), only one missense mutation in the TM domain (*MFN2*^R632W^) in the Genome Aggregation Database (gnomAD) has been identified by ClinVar as potentially pathogenic [45]. This allele was identified by exome sequencing of a Japanese family in which two of three daughters from consanguineous parents were diagnosed with a late-onset, recessive form of CMT. In addition to common CMT symptoms such as distal muscle atrophy, peripheral axon degeneration, and areflexia, the patients displayed decreased cerebellar perfusion and atrophy of the cerebellum and thoracic spinal cord [45]. The mouse line reported here (*Mfn2*^L643P^) is the first murine model with a pathogenic mutation in the TM domain [13,19,23,24,25,26,37]. Despite overt gait and behavioral deficits consistent with neuromuscular disease, further neurophysiological and histological analysis of peripheral nerves from these mice did not reveal signs of peripheral neuropathy. However, defects in skeletal muscle, fertility, and bone formation were observed. The diverse range of symptoms and atypical location of the mutation should make this mouse line of interest to researchers studying the biological functions of MFN2.

## 2. Materials and Methods

### 2.1. Generation and Maintenance of the MFN2 Mutant Line

All procedures were performed according to the guidelines of the National Institutes of Health Guide for the Care and Use of Laboratory Animals and were approved by the Institutional Animal Care and Use Committees of the Genomics Institute of the Novartis Research Foundation (GNF), UNC-Chapel Hill, and The Jackson Laboratory.

Eight-week-old male C57BL/6J mice (Generation G0) were injected intraperitoneally once a week for three weeks with 100 mg/kg N-ethyl-N-nitrosourea (ENU; Sigma-Aldrich, St. Louis, MO, USA) freshly dissolved in 10% ethanol in citrate buffer, pH 5.0. They were then mated to wild-type (WT) C57BL/6J (B6) females to produce G1 offspring, each with one copy of the mutations inherited from the G0 sire. G1 animals were analyzed for visible phenotypes of dominant and semi-dominant traits. G1 males were crossed to wildtype B6 females to produce G2 animals, and the G2 females were bred back to the G1 sire to yield G3 offspring. These G3 animals were screened for specific, postnatal abnormalities such as congenital malformations, clinical chemical, biochemical, hematological and immunological/allergological defects, and behavioral deficits to identify recessive traits of interest. Prior to the identification of the *Mfn2* mutation, the line was maintained by in vitro fertilization (IVF) to B6 females using sperm from affected animals. After the identification of the mutation, the line was maintained by heterozygous matings. Genotyping was conducted by PCR using the following primers, followed by sequencing the amplicon using the reverse primer: FWD PRIMER—5′ CAG GTG TGG AAG GCA GTG 3′; REV PRIMER—5′ TCA CTG CTG GAC TTG GTG 3′. All mice were group housed (2–5 animals per cage) in ventilated cage racks in a temperature-controlled animal room (22 °C) with a 12 h/12 h light/dark cycle. Water and food were provided ad libitum.

### 2.2. Single Nucleotide Polymorphism (SNP) and Microsatellite Mapping

A total of 77 B6/FVB and 180 B6/DBA F2 progeny were genotyped with a panel of 289 genome-wide single nucleotide polymorphisms (SNPs) using matrix-assisted laser desorption ionization-time-of-flight single-base extension reactions. After SNP alignment, a region on the distal end of chromosome 4 was found to segregate with the phenotype, with the exception of only two mice that were affected, but heterozygous in this region. Subsequent fine mapping was carried out using a panel of 22 additional SNPs spanning the region between 126.4 and 153.3 Mb on chromosome 4, as well as the MIT microsatellite markers D4MIT343, D4MIT160, D4MIT285, D4MIT127, D4MIT33 and D4MIT42. This narrowed the region of interest to 5 Mb between 143.64 and 148.769 Mb, which included two candidate genes—*Kif1B* and *Mfn2*, both of which are associated with neurological phenotypes. Additional genes in the interval are listed in Table 1. All 51 exons of the *Kif1B* gene, 22 exons of the *Mfn2* gene, as well as 250 bp of adjacent introns, were sequenced using genomic DNA from two affected animals and two unrelated B6 animals. All genomic coordinates refer to NCBI Mouse Genome build 36 unless otherwise stated. 

### 2.3. Tissue Preparation for Histology and Electron Microscopy

Cardiac perfusion was performed on mice, first with 0.1 M phosphate buffer, pH 7.4, followed by a solution of 4% paraformaldehyde (PFA), and 2.5% glutaraldehyde in phosphate buffer until the mice became stiff. Brains, tibialis anterior muscle (TA), and femoral nerves were dissected and processed for histology and transmission electron microscopy (TEM). Brains were sectioned at 10 μm and mounted on Superfrost Plus slides (Fisher). Matched sagittal sections were stained with H&E and digitally imaged using a Nikon Microphot FXA, (Nikon, Inc., Garden City, NY, USA) and a QImaging Micropublisher 3.3 CCD Digital Camera (QImaging Corporation, Surrey, BC, Canada). The motor and sensory branches of the femoral nerve were plastic-embedded, sectioned at 0.5 μm thickness, and stained with toluidine blue to visualize myelin. These slides were imaged on a Nikon 360 NoScope with a 20X objective. Ultrathin sections of TA were mounted on grids for TEM and imaged with LEO EM 910 Transmission Electron Microscope. Samples examined for signs of pathology including abnormal, thin, or missing myelination, degenerating and/or regenerating axons, abnormalities in the axoplasm or cell bodies such as the presence of vacuoles, disrupted cytoskeletal components or organelles, with particular attention paid to abnormalities in mitochondria shape or localization. Axon number, nerve area, and mitochondrial diameter were measured and compared between genotypes.

### 2.4. Immunofluorescence

Triceps surae and spinal cords were immersion fixed in fresh 2% PFA in 1X PBS overnight at 4 °C, cryopreserved in 30% sucrose in 1X PBS overnight, and frozen in Tissue-Tek OCT compound (Sakura Finetek USA, Torrance, CA, USA) by immersion in a slurry of isopentane and dry ice. Specific immunofluorescence procedures were as follows:

#### 2.4.1. Spinal Cord Motor Neurons

Spinal cords were cross-sectioned (15 µm), blocked in 2% BSA in PBS with 0.2% Triton X-100 for 2 h at room temperature (RT), then incubated for 48 h in goat anti-ChAT (1:100, 315-Chat from Phosphosolutions, Aurora, CO, USA). Sections were washed three times for 30 min each in PBS with 0.2% Triton X-100 and incubated overnight in AlexaFluor647-conjugated bovine anti-goat secondary antibody (705-605-147 from Jackson Immunoresearch, West Grove, PA, USA) diluted in blocking solution. After washing three more times, sections were mounted with Prolong Gold antifade (ThermoFisher, Waltham, MA, USA) and imaged using a Leica Stellaris 8 WLL confocal microscope with a tunable supercontinuum laser and HyD photon-avalanche detectors.

#### 2.4.2. Muscle Fiber Typing

Muscle fiber-type staining was conducted with modifications to a protocol from the University of Kentucky Muscle Biology Center [46]. Briefly, 15 µm cross sections were cut through the triceps surae and allowed to dry overnight at room temperature. Sections were rehydrated with three washes of 1X PBS, then incubated overnight at 4 °C with primary antibodies: mouse anti-MYH7 for type 1 fibers (1:100, BA.D5-c from Developmental Studies Hybridoma Bank (DSHB), Iowa City, Iowa) and mouse anti-MYH2 for type IIa fibers (1:100, SC.71-c from DSHB). Sections were washed three times in 1X PBS, then incubated for 2 h at RT with secondary antibodies: AlexaFluor647-conjugated goat anti-mouse IgG2b for type 1 fibers (1:250, A21242 from ThermoFisher) and AlexaFluor488-conjugated goat anti-mouse IgG1 for type IIa fibers (1:250, A21121 from ThermoFisher). Sections were washed three more times in 1X PBS, post-fixed for 5 min in methanol, washed three more times in 1X PBS, and mounted with Prolong Gold Antifade (ThermoFisher). Muscle sections were imaged using a Leica DMi8 widefield fluorescent microscope.

#### 2.4.3. Neuromuscular Junction Staining

Longitudinal sections (50 µm) were cut through the triceps surae, blocked for 1 h in 2% BSA in PBS with 0.2% Triton X-100, incubated overnight in primary antibodies: mouse anti-NEFM (1:250, 2H3-c, DSHB) and mouse anti-SV2A (1:250, SV2-c, DSHB). This was followed by three 2 h washes in PBS with 0.5% Triton X-100 and 2h incubation with AlexaFluor488-conjugated goat anti-mouse secondary antibody (1:500, 715-545-150 from Jackson ImmunoResearch) and AlexaFluor555-conjugated α-bungarotoxin (1:1000, B35451 from ThermoFisher) to label post-synaptic acetylcholine receptors. NMJs were imaged using a Leica Stellaris 8 WLL confocal microscope.

### 2.5. Western Blotting

To observe MFN2 protein levels, total protein was extracted from the medial gastrocnemius of WT and homozygous mutant littermates by dounce homogenization in RIPA buffer with 1X Turbocharged protease and phosphatase inhibitor cocktail (T-2495 from Ag Scientific, San Diego, CA, USA). The homogenate was centrifuged for 15 min at 13,000× *g* and the total protein concentration of the supernatant was measured using a bicinchoninic acid (BCA) assay (23227 from ThermoFisher); 10 µg total protein was loaded on Bolt 4–12% Bis-Tris gels (NW04122BOX from ThermoFisher), transferred to PVDF membrane, blocked with 3% bovine serum albumin in tris-buffered saline (TBS) with 0.1% Tween-20, and probed overnight with rabbit anti-MFN2 antibody (1:10,000; ab124773 from Abcam, Cambridge, UK) or rabbit anti-GAPDH (1:10,000; 2118 from Cell Signaling Technologies, Danvers, MA, USA). After washing, the blot was incubated with HRP-conjugated goat anti-rabbit or anti-mouse secondary antibody (1:125,000; Jackson Immunoresearch). Proteins of interest were visualized by incubating the blot with a chemiluminescent substrate and exposing it to X-ray film. The protein bands were quantified using ImageJ software. MFN2 levels were normalized to GAPDH levels.

### 2.6. Analysis of mtDNA/nDNA Ratio

To determine the ratio of mitochondrial DNA (mtDNA) to nuclear DNA (nDNA), a protocol was adapted from Quiros et al., 2017 [47]. DNA was isolated from snap frozen lateral gastrocnemius muscle using the Qiagen DNEasy Blood and Tissue Kit (69504 from Qiagen, Hilden, Germany). The mitochondrial genes, ND1 (gene symbol: *Mtnd1*) and 16S rRNA (gene symbol: *Mtrnr2*), were compared to the nuclear gene, *Hk2*. The following primer pairs were used: ND1 F: 5′-CTAGCAGAAACAAACCGGGC-3′ R: 5′-CCGGCTGCGTATTCTACGTT-3′, 16S rRNA F: 5′-CCGCAAGGGAAAGATGAAAGAC-3′ R: 5′-TCGTTTGGTTTCGGGGTTTC-3′, and *Hk2* F: 5′-GCCAGCCTCTCCTGATTTTAGTGT-3′R: 5′-GGGAACACAAAAGACCTCTTCTGG-3′. All qPCR reactions were carried out in quadruplicate in a 384-well plate. Analysis was then carried out in two different ways to ensure confidence in the results. The ΔΔCt method was used to calculate the relative expression of each of the samples. ΔCt was calculated using the following equation: ΔCt = Ct(mtDNA gene) − Ct(nDNA gene). ΔΔCt was then calculated using the mean of the wildtype cohort as the control sample: ΔΔCt = ΔCt(Sample of interest) − ΔCt(Control Sample). Finally, the expression of each sample was 2^−ΔΔCt^. To determine the number of copies of mtDNA, ΔCt was first calculated with the following formula: ΔCt = Ct(nDNA gene) − Ct(mtDNA gene). Then, the mtDNA copy number was determined with mtDNA = 2 × 2^ΔCt^. Finally, the relative mtDNA content is then calculated with Relative mtDNA content = mtDNA(affected)/mtDNA(control). Data were imported into GraphPad Prism for statistical analysis.

### 2.7. Open-Field Exploration

The open field test is a simple assessment used to determine general activity levels, gross locomotor activity, anxiety, and exploratory behavior in mice. The open field apparatus was a 17″ × 17″ × 13″ arena with a white Plexiglas floor and clear Plexiglas walls (ENV-515, Med Associates Inc, Fairfax, VT, USA) surrounded by infrared detection beams on the X, Y and Z-axes that track the animals’ position and activity over the course of the experiment. The apparatus is isolated within a 73.5 × 59 × 59 cm testing chamber fitted with overhead fluorescent lighting (Lux level 14). Following a short acclimation period in a quiet anteroom, mice were removed from their home cage, immediately placed in the corner of the open field arena and allowed to freely explore the apparatus for a 10 min recorded test interval. Mice were scored using Activity Monitor (v5.1, Med Associates Inc.) for total distance traveled and the number of rearing events. Between each trial, the open field arena was cleaned with a 0.1% bleach solution and allowed to dry completely.

### 2.8. Inverted Wire Hang Test

As a behavioral measure of muscular endurance, we utilized the inverted wire hang test [48,49,50]. In this assay, mice were placed on an inverted wire grid for a maximum trial time of two minutes and latency for mice to fall was measured. Mice that fell from the grid within the first minute of the test were returned to it a maximum of two more times and the longest time they remained on the grid was recorded. This was repeated five consecutive times within the same session and the highest value from the five trials was recorded. Mice were tested every two weeks between 4 weeks and 16 weeks of age, though homozygous mutant animals were not tested past 12 weeks due to their inability to perform the task. Mice were not trained prior to the initial test session.

### 2.9. Rotarod Assay

Beginning at six weeks, motor coordination was also tested in a simplified rotarod assay. Seventeen animals per genotype were tested every two weeks between 6 weeks and 16 weeks of age (homozygous mutant animals were not tested past 12 weeks due to inability to perform the task). A relatively low, fixed speed of 16 rpm and a two-minute testing window was used. Prior to the first test at six weeks of age, mice were trained on the rotarod for two minutes on three consecutive days, with the final training session taking place two days prior to the testing day. Mice were placed onto the rotating rod and latency to fall from the rod was measured. Mice that failed to remain on the rod were returned to the apparatus a maximum of two more times and the longest time they remained was recorded.

### 2.10. Mechanosensitivity and Thermosensitivity Test

Because neurosensory deficits are one of the hallmarks of human CMT2A, we also examined the touch and temperature sensitivity of *Mfn2*^L643P^ animals. The tests were conducted at eight weeks of age when ataxia was not visibly observed but subtle deficits were detectable in the open field and wire hang tests. The mechanosensory response was measured in a plexiglass chamber with a wire grid floor using a dynamic plantar aesthesiometer (model 37400 from Ugo Basile, Gemonio VA, Italy) comprised of a moveable touch-stimulator unit with a 0.5 mm rigid Von Frey filament and force actuator. Mice were allowed to acclimate to the testing environment for two hours, then the aesthesiometer was positioned beneath the plantar region of the animal’s rear paw. At a start signal, the filament rose with a preset force increase until either the animal withdrew its paw, or a preset maximum force was reached. Two parameters were recorded, force in grams required to elicit the paw withdrawal response and latency to withdraw in seconds. Both right and left rear paws were tested a total of four times, with at least one-minute rest intervals between tests, and measurements were averaged to give a mean value for each paw.

Two days following the mechanosensory test, thermosensitivity was assessed using the hot plate assay. In this test, twenty-one mice per genotype were placed on a preheated 55 °C plate surrounded by a cylindrical Plexiglas wall (Ugo Basile) and closely observed for response to heat as indicated by flicking or licking any paw or attempting to jump out of the apparatus. At this point, the mouse was removed from the apparatus and the animal’s latency to respond was recorded. Animals that failed to respond were removed at one minute, and each animal was tested only once.

### 2.11. Electromyography

Mice were anesthetized with 2% isoflurane and placed on a thermostatically regulated heating pad to maintain body temperature. Three recording electrodes were placed subcutaneously as such: earth and (+) in the plantar region of the left and right hindpaw, respectively, and (−) between the last two digits of the right hindpaw. Nerves were stimulated distally at the ankle, then proximally at the sciatic notch. Compound muscle action potential amplitude (CMAP), which is the summation of action potentials from flexor digitorum brevis muscles in the plantar hindpaw, and latency to CMAP peak from each stimulus were recorded. NCV was calculated as [conduction distance/(proximal latency-distal latency)].

### 2.12. In Vivo Contractile Function (Ankle Dorsiflexion)

An integrated system (Aurora Scientific, Toronto, ON, Canada) was used for the measurement of ankle dorsiflexion forces. Each mouse was prepared as previously described [51], except that stimulation was delivered directly to the dissected and transected tibial nerve via a silver bipolar electrode on which the nerve was placed. All experiments were terminal.

Briefly, mice were induced in a chamber with 5% isoflurane (1 L/min O_2_ flow) and then maintained, via nose cone, with 2% isoflurane for the duration of the experiment. Once anesthetized, a small incision (~1 cm) was made to expose and free the peroneal nerve on the medial side of the right leg for later placement on the stimulating electrode. Next, the animal was placed supine on a heated platform (37 °C), and a rectal probe was inserted to allow body temperature to be monitored and maintained. The right foot was taped securely to the footplate on the transducer using medical tape and the right knee was clamped to immobilize the leg during stimulation during force measurements. The platform was then adjusted, as needed, to establish a 90-degree angle at the ankle joint. Finally, the peroneal nerve was transected and the distal end was placed gently on the electrode. The nerve was covered with a small piece of cotton soaked in warmed mineral oil to protect it from drying out.

Stimulus Protocols: Dorsiflexion force was recorded in response to the following stimuli: (i) a single 0.2 ms pulse (twitch force, Pt), (ii) a 15 pulse train, 20 ms interpulse interval (50 Hz submaximal tetanic contraction) and, (iii) a 90 pulse train, 6.7 ms interpulse interval (150 Hz maximal tetanic contraction). Fatigue was assessed by eliciting repetitive submaximal contractions for 10 min (6-pulse, 60 Hz trains, delivered at 0.5 Hz) Fatigue was calculated as final peak force/initial peak force. Data were captured and analyzed using Aurora Scientific software (DMD and DMA, respectively).

### 2.13. MicroCT Analysis

The micro-architecture of the trabecular bone and cortical bone of the femur and tibia were analyzed by µCT (resolution 10.5 µm, VivaCT-40, Scanco Medical AG, Bruttisellen, Switzerland). Bones were scanned at an energy level of 70 kVp, a tube current of 114 µA, and an integration time of 200 ms. Measurements of the trabecular bone included bone volume/total volume (BV/TV), trabecular number (Tb.N.), trabecular thickness (Tb.Th.) and trabecular separation (Tb.Sp.) and for the cortical bone total cross-sectional area (Tt.Ar), cortical bone area (Ct.Ar.), medullary area (Ma.Ar.), bone area fraction (Ct.Ar/Tt.Ar.), cortical tissue mineral density (Ct.TMD.), cortical thickness (Ct.Th.) cortical porosity (%), and maximum, minimum, and polar moments of inertia (Imax, Imin, and pMOI). Trabecular bone volume fraction and micro-architecture of the femur were evaluated in the secondary spongiosa, starting proximately at 0.21 mm proximal to the distal femoral growth plate, and extending proximally 1.575 mm (150 slices). Scans for the cortical region were measured at the mid-point of each femur and extended distally 0.525 mm (50 slices). In the tibia, the trabecular bone region of interest started 105 µm (10 transverse slices) distal to the break in the growth plate and extended 1050 µm (100 transverse slices). The cortical bone region of interest started 1995 µm proximal to the fibular junction and extended 525 µm (50 transverse slices) distal. Bone was segmented from soft tissue using a mineral density threshold of 300 mg HA/cm^3^ for trabecular bone and 700 mg HA/cm^3^ for cortical bone. All scans were analyzed using manufacturer software (Scanco, version 4.05). Acquisition and analysis of uCT data were performed in accordance with published guidelines [52].

Statistical analyses were performed using GraphPad Prism 9. Data were not tested for normality as the sample sizes were generally too low for this to be meaningful. Outliers were not excluded, and all data points were included in the analysis.

## 3. Results

### 3.1. ENU Mutagenesis, Mapping, and Expression

A 20-week-old G3 male from ENU mutagenesis line 715 exhibited priapism, weight loss and progressive deterioration of motor coordination, characterized by decreased locomotion with a wobbly, ataxic gait. This seemed like a potentially interesting model of neuromuscular disease, so an attempt was made to establish this mutant line using sperm from this male for in vitro fertilization (IVF). Although the sperm collected from this male appeared morphologically normal and motile, the IVF failed and no offspring were produced. The line was eventually recovered by breeding three phenotypically normal male G3 siblings (presumed WT or heterozygous for the mutation) to WT C57BL/6J females and then crossing the resulting females back to their sire and observing these offspring for signs of locomotor dysfunction. Affected mice were identified and sperm from an affected male was successfully bred using IVF to establish the line. The offspring from this IVF were intercrossed to yield 117 animals in the F2 generation, of which twenty-nine (24.8%) eventually showed the phenotype, with approximately equal numbers of males and females affected. This pattern of inheritance has remained constant in all subsequent crosses; thus, we concluded that the mutation acts in a purely recessive manner, penetrance is complete and there is no embryonic lethality associated with it. Impaired gait and neuromuscular performance were evident by 8 weeks of age and progressed with age (See Supplemental Movies of 8, 12, and 16-week mice).

**Figure 1:**
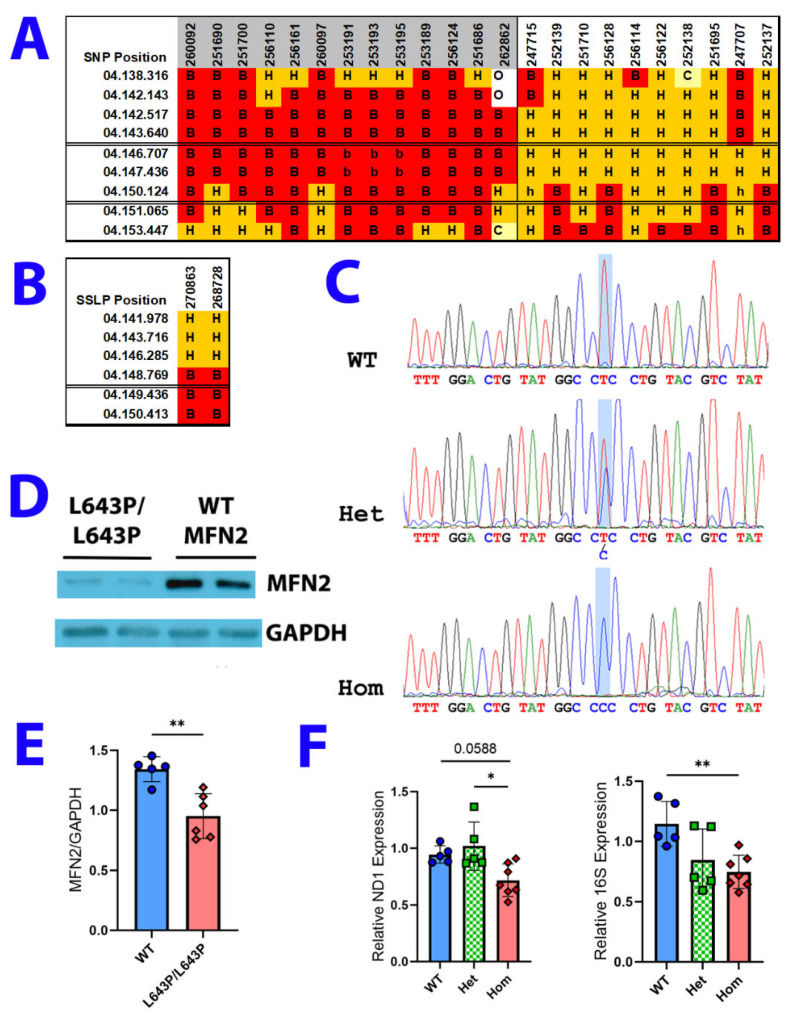
ENU-induced mutation maps to chromosome 4. (**A**,**B**) SNP and SSLP genotyping localize the mutation in ENU line 715 to chromosome 4 between 143.640 and 148.769 Mb (NCBI Build 36). ID numbers of affected animals are shaded gray, unaffected animals are unshaded. Marker positions are at the left in each table. B6 homozygosity is indicated by a B, heterozygosity by an H and DBA or FVB homozygosity by a C. O represents missing data. (**C**) Mutation identified in exon 18 of the *Mfn2* gene. T to C transition at bp 1928 introduces a Leu to Pro substitution at amino acid 643, which resides in the transmembrane domain of the protein. (**D**) Western blot showing decreased levels of MFN2 protein in medial gastrocnemius of 16-week-old WT and homozygous mutant mice. (**E**) Quantification of MFN2 Western blots normalized to GAPDH levels. N = 5 WT, 6 homozygous mutant mice. (**F**) qPCR analysis shows a small but not statistically significant (*p* = 0.0588) decrease in the mitochondrially encoded ND1 gene and significantly reduced levels of the mitochondrial 16S ribosomal RNA in homozygotes compared to WT mice. N = 7 homozygous mutants, 5 heterozygous mutants, and 5 WT mice. Error bars indicate SD. *, ** *p* < 0.05, and 0.01 by unpaired *t*-test or one-way ANOVA.

To identify the gene responsible for the phenotype in this mutant line, we initiated two separate mapping crosses to DBA/2J (DBA) and FVB/NJ (FVB) inbred mouse strains with two affected males on a C57BL/6J background. Mapping F2 mice from these crosses using single nucleotide polymorphisms (SNPs) identified a ~5 Mb interval between 143.64 and 148.769 Mb (NCBI Build 36; 144.1 and 150.01 Mb in Build 39) on Chromosome 4 as the causative locus. (Figure 1A,B). Of the 257 F2 animals from the mapping cross that were genotyped, only two conflicts were identified, both apparently affected animals that were heterozygous throughout the region. 

Among the genes in this 5 Mb interval (Table 1), we identified two excellent candidate genes—Kinesin Family Member 1B (*Kif1B*) and Mitofusin 2 (*Mfn2*) located at 148.020 and 146.717 Mb, respectively (149.261 and 147.958 Mb in Build 39). Mice lacking *Kif1B* have a neuromuscular phenotype and mutations in *MFN2* cause CMT2A [18,33,53]. All exons and 250 bp of adjacent 5′ and 3′ introns from both genes were sequenced using genomic DNA from two affected mice and two WT C57BL/6J mice. No polymorphisms were found in the *Kif1B* gene, but an SNP was found in exon 18 of the *Mfn2* gene, a T to C transition at base 1928 changing amino acid 643, leucine, to proline (Figure 1C). This amino acid change is within a predicted α-helix in the transmembrane domain and has the potential to significantly perturb secondary structure and protein function.

To determine if this mutation affects protein expression, Western blots were performed using the lateral gastrocnemius muscle. MFN2 protein levels were normalized to levels of GAPDH. Homozygous mutant mice had decreased MFN2 protein levels compared to WT controls (Figure 1D,E). Since mitochondrial fusion is a key regulator of mitochondrial DNA integrity, we quantified mtDNA content in the lateral gastrocnemius as a marker for mitochondrial health. To do this, we performed qPCR for two mitochondrial-encoded genes, NADH Dehydrogenase 1 (ND1) and 16S rRNA, and normalized them to levels of the nuclear-encoded gene hexokinase 2 (HK2). In homozygous *Mfn2* mutants compared to WT controls, levels of 16S rRNA were lower by ~35% (*p* = 0.009) and ND1 levels by ~24% (*p* = 0.0588). Interestingly, while 16S rRNA levels were ~26% lower in heterozygous *Mfn2* mutants compared to WT (*p* = 0.0663), ND1 levels were slightly lower in WT (~7.5% less; *p* = 0.7173) and significantly lower in homozygous mutants (~30% less; *p* = 0.0117) compared to heterozygous mice (Figure 1F)

While all genotypes occurred in the expected ratios of 25% WT, 50% heterozygous and 25% homozygous, we noticed that using sperm from *Mfn2* mutant males for IVF resulted in a lower oocyte fertilization rate (5.7% vs. 65% when using WT B6 sperm), fewer pups produced (14.1% of 2-cell embryos vs. 50% for WT B6), and a relatively low rate of successful IVF (see Table 2). Furthermore, we are confident that the mutation we identified is the causative locus and the official designation for the allele is *Mfn2*^m1Lmt^, but will be referred to as *Mfn2*^L643P^, or simply L643P, throughout the rest of the manuscript. However, it should be noted that this mutant resulted from a genome-wide mutagenesis strategy. As a result, it is possible that the mice harbor additional mutations, some of which may affect the coding sequence, although only closely linked mutations on Chr. 4 would co-segregate after multiple outcrosses to WT mice. Null alleles of the *Mfn2* gene lead to embryonic lethality [32]. Since no such lethality is observed in homozygous *L643P* animals, we predict that this is not a complete loss of function allele, but rather a hypomorph. 

### 3.2. Changes in Body Weight and Behavior

**Figure 2:**
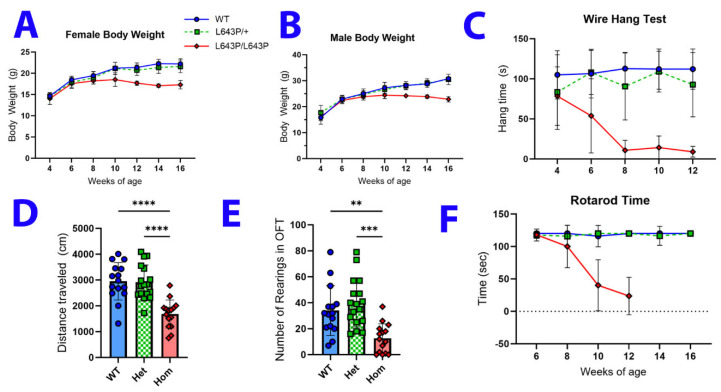
*Mfn2*^L643P/L643P^ mice have neuromuscular deficits. (**A**,**B**) Body weights of female and male mice were collected from 4 to 14 weeks of age. N = 10 female and 7 male mice per genotype. (**C**) Homozygous *Mfn2* mutants show a significant deficit in the wire hang test of muscular endurance by 6 weeks of age, compared to heterozygous and wildtype littermates. There are between 6 and 44 mice per genotype at each time point, with a roughly equal mix of males and females. (**D**,**E**) In an open field test at 8 weeks of age, affected mice traveled less total distance and had fewer rearing events compared to unaffected littermates. N = WT (9F/6M), Het (10F/7M), Hom (10F/5M). (**F**) Rotarod data show that homozygous *Mfn2* mutant mice have decreased latency to fall compared to unaffected mice beginning at 8 weeks. N = 10F/7M per genotype. Error bars indicate SD. **, ***, **** *p* < 0.01, 0.001, and 0.0001, respectively.

Animals were weighed every other week from four to sixteen weeks of age. At 10 weeks, a significant difference in body weight was noted between the mutants and controls, and this progressed through 16 weeks, at which point most mutant mice were losing weight (*p* < 0.0001 both males and females) (Figure 2A,B). No significant weight difference was detected between heterozygous animals and WT littermates. Furthermore, aside from a decrease in grip strength and endurance beginning at six weeks, homozygotes are visibly indistinguishable from littermates until approximately eight weeks of age. 

To measure grip strength and endurance, mice were placed on an inverted wire grid from which they must hang for a maximum of two minutes. There was no significant difference in this task until six weeks of age when the mean latency to fall from the grid for homozygous mutants is approximately half that of heterozygous and WT animals (*p* < 0.0001). By eight weeks, their mean latency to fall drops to 11 s and remains approximately the same thereafter (*p* < 0.0001 for weeks eight through twelve, Figure 2C). Mutant animals were not tested beyond 12 weeks due to their inability to perform the test. However, heterozygous and WT animals were tested until 16 weeks of age and only the eight-week timepoint showed a small, but significant difference between the genotypes, which is likely a testing anomaly.

At eight weeks of age, affected mice show decreased locomotor activity and rearing events in the open field. A total of 47 animals were tested and 15 of these animals subsequently exhibited the motor phenotype. The affected animals show a 43% decrease in overall distance traveled during the ten-minute test (*p* = 0.0001 when compared to hets, *p* = 0.003 compared to WT) and a 63% decrease in the number of rearing episodes compared to unaffected littermates (*p* < 0.0001 compared to both hets and WT mice) (Figure 2D,E). Decreased locomotor activity is quickly followed by a decline in motor coordination. At eight weeks there is a noticeable, but not quite significant (*p* < 0.07) decrease in the latency to fall on the rotarod test, which progresses to statistical significance by 10 weeks and drops further at 12 weeks (Figure 2F). Mutants were not tested past twelve weeks because their coordination is so impaired that they are unable to stay on the rotarod for any length of time. No difference was observed between heterozygous and WT littermates at any timepoint through 16 weeks.

These data agree with other behavioral phenotypes that were observed, but not quantified. For example, by approximately 10 weeks of age, movement in both males and females became clumsy and unsteady (see Supplementary Movies of male mutant mice and unaffected littermates). This progressed such that normal activities including locomotion, breeding, grooming and feeding are impaired (movies). By 20 weeks of age, affected mice exhibited very little voluntary movement, were strikingly uncoordinated when they did move, and exhibited increasing morbidity and mortality. We have not aged affected animals to precisely define mortality associated with this mutation due to the precipitous decline in health they exhibit once the locomotor phenotype presents.

### 3.3. Neuromuscular Physiology and Sensory Testing

**Figure 3:**
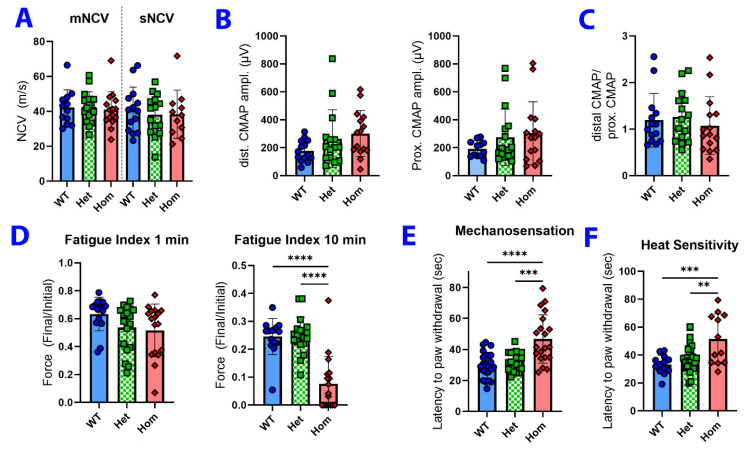
Assessing peripheral motor and sensory nerve physiology for signs of CMT2A. (**A**) Motor and sensory nerve conduction velocities (mNCV and sNCV) were not significantly different between genotypes. N for mNCV = WT (8F/6M), Het (8F/8M), Hom (6F/9M). N for sNCV = WT (8F/6M), Het (7F/8M), Hom (4F/7M). (**B**) Distal and proximal compound muscle action potential (CMAP) amplitudes were all unchanged across genotypes. (**C**) The ratio of distal/proximal CMAP, an indicator of possible conduction block, was also unchanged across genotypes. For B-C, N = WT (8F/6M), Het (7F/8M), Hom (9F/6M). (**D**) Fatigue index (force of final muscle contraction/force of first contraction) of tibialis anterior (TA) muscle shows greater fatigue in homozygous mutant mice compared to WT or heterozygous mice after 10 min stimulation, but not after 1 min. N = WT (8F/7M), Het (9F/10M), Hom (10F/7M). (**E**,**F**) Homozygous mutants showed decreased mechanosensation and heat sensitivity. N = WT (8F/6M), Het (15F/8M), Hom (11F/9M). Error bars indicate SD. ** *p* < 0.01; *** *p* < 0.001; **** *p* < 0.0001 by one-way ANOVA.

To assess motor nerve function, electromyography (EMG) was conducted to calculate the sciatic motor and sensory nerve conduction velocity (NCV) and distal/proximal compound muscle action potential (CMAP) amplitude. No significant changes in these measures were seen between genotypes (Figure 3A,B). CMAP amplitudes elicited from proximal stimulation (sciatic notch at the hip) and distal stimulation (ankle) were also compared to detect possible conduction block (distal amplitude > proximal amplitude) but no changes were seen (Figure 3C). However, mutants had a lower fatigue index (increased fatigue) compared to WT mice after 10 min of repetitive tibialis anterior (TA) muscle contractions, indicating impaired muscular endurance (Figure 3D, Table 3). Because neurosensory deficits are one of the hallmarks of human CMT2A caused by *Mfn2* mutations, we also examined the touch and temperature sensitivity of *Mfn2*^L643P^ animals. Interestingly, homozygous *Mfn2*^L643P^ mutants took more time than control animals to react to a wire filament poke or an elevated temperature stimulus in the Von Frey test and hot plate assay, respectively (Figure 3E,F). This finding indicates decreased sensitivity to mechanical stimulus and heat. No significant difference was observed between heterozygotes and WT littermates. However, we cannot rule out that the motor deficit present in homozygous *Mfn2*^L643P^ mice may have confounded the paw withdrawal reflex in these tests. 

### 3.4. Histopathology

**Figure 4:**
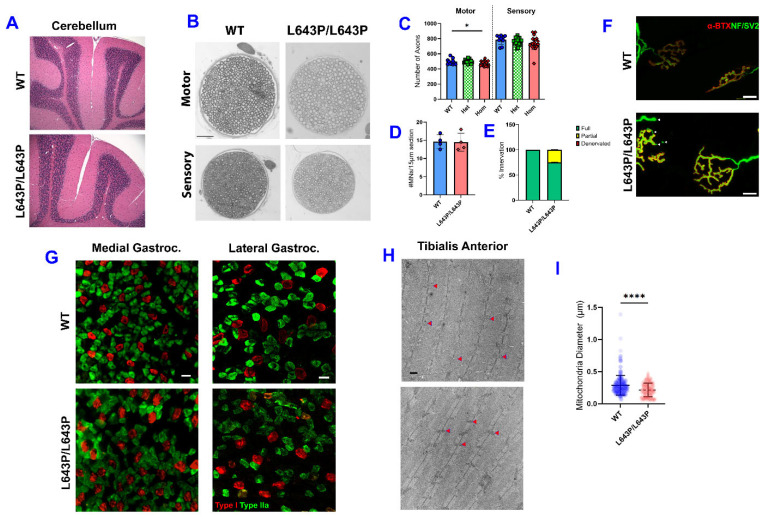
Histological assessment of *Mfn2*^L643P^ mouse nervous system and skeletal muscle. (**A**) Gross cerebellar morphology is unaffected by the mutation (affected mouse on bottom). (**B**) Representative 20× images of cross-sections of the motor and sensory branches of the femoral nerve stained with toluidine blue. (**C**) Quantification of axon number in each branch of the femoral nerve. N = WT (8F/5M), Het (8F/9M), Hom (12F/9M) (**D**) Quantification of the number of ChAT+ motor neurons per 15 µm spinal cord section. (**E**) Percentage of NMJs that are fully innervated (green), partially innervated (yellow), and denervated (red). (**F**) Representative confocal images of WT and homozygous mutant NMJs. Both WT NMJs and the mutant NMJ on the right are fully innervated, while arrowheads indicate partial denervation. Scale bar = 10 µm. (**G**) Representative immunofluorescent images showing a lack of fiber type grouping in the medial and lateral gastrocnemius in WT and mutant mice. Red—Type 1 (slow) fibers, Green—Type 2a fibers. Scale bar = 50 µm, for (**D**–**G**), N = WT (2F/2M), Hom (4M). (**H**) Representative 1500X TEM images showing mitochondria in tibialis anterior muscle in unaffected (top) and affected (bottom) animals. Some mitochondria are indicated by red arrowheads. (**I**) Diameter of mitochondria in TA of affected (yellow) and unaffected (red) mice. For H-I, N = 2F/2M per genotype. * *p* < 0.05; **** *p* < 0.0001 by one-way ANOVA or unpaired *t*-test.

To evaluate if the loss of motor coordination is due to defects in the CNS, such as Purkinje cell degeneration or central demyelination, a preliminary histological assessment of the whole brain at 12 weeks was conducted. No gross abnormalities were observed in cortical, midbrain, or brainstem structures (data not shown). Additionally, no gross differences between affected and unaffected littermates were detected in the Purkinje, granule, or molecular layers in the cerebellum, suggesting that cerebellar neurodegeneration is not the cause of the ataxic movement observed in this line (Figure 4A). It is possible that subtle defects or lesions are present in the affected mice that we did not detect. We next examined the motor and sensory branches of the femoral nerve to determine if the mice had peripheral neuropathy. No overt signs of axon degeneration, demyelination, or other indications of peripheral neuropathy were observed by light microscopy (Figure 4B). However, the number of myelinated axons in the motor branch was slightly but significantly reduced (*p* = 0.045) in homozygous mutant mice compared to unaffected littermates, while the sensory branch was unchanged (Figure 4C). To determine if this correlated with a loss of spinal cord motor neurons, lumbar spinal cord sections were labeled with an antibody to choline acetyltransferase (CHAT) and motor neurons were counted, revealing no differences in number of motor neuron cell bodies (Figure 4D). Neuromuscular junctions were evaluated and while there was a slight increase in the number of partially innervated NMJs, this did not reach significance and no signs of denervation were seen (Figure 4E,F). 

No evidence of muscle fiber type grouping or switching was seen in cross-sections of the medial and lateral gastrocnemius (Figure 4G). Since a decrease in muscular endurance was seen in the TA (Figure 3D), TEM was performed on the same muscle and the diameters of 227 WT and 233 *Mfn2*^L643P/L643P^ mitochondria were measured from four mice per genotype. This revealed a significant reduction in the average mitochondrial diameter in mutant mice compared to WT (Figure 4H,I). However, there was a large variation in mitochondrial diameter in both genotypes, so it was not possible to distinguish the groups simply by looking at images of their mitochondria.

**Figure 5:**
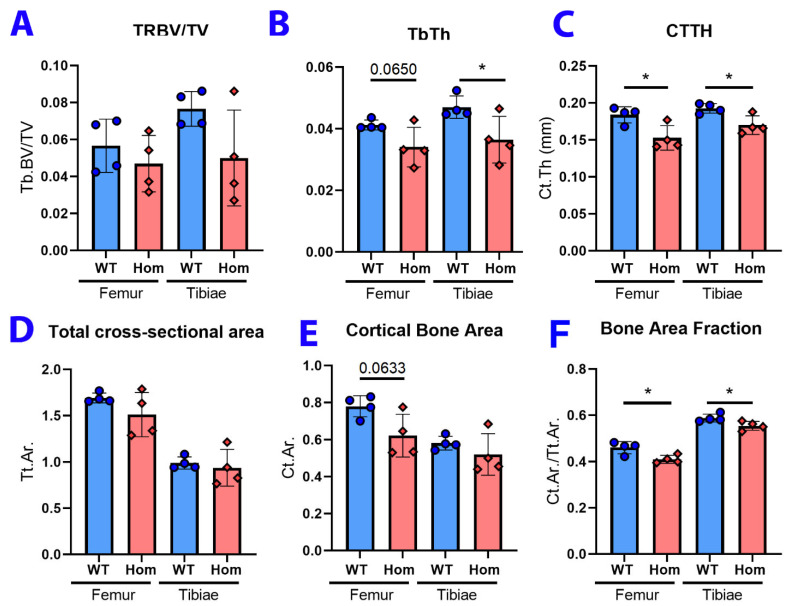
MicroCT analysis of leg bones from *Mfn2*^m1Lmt/m1Lmt^ mice. (**A**) Trabecular bone volume fraction was not significantly different between genotypes in the femur or tibia. (**B**) Trabecular bone thickness was significantly decreased in mutant mice compared to controls. (**C**) Cortical thickness of the femur and tibia of mutant mice was significantly decreased. (**D**,**E**) Total cross-sectional area (Tt.Ar) and cortical bone area (Ct.Ar.) were unchanged between genotypes. (**F**) Bone area fraction (Ct.Ar/Tt.Ar.) was decreased in both the femur and tibia of mutant mice. N = 4 female mice per genotype for all microCT experiments. * *p* < 0.05. *p*-values are shown on graphs for values near the 0.05 cutoff when analyzed by paired *t*-test.

Because decreased neuromuscular loading can affect the bone thickness and density, and recent work shows that *Mfn2* plays a role in bone development [35,36,54], microCT analysis was performed on the tibiae and femurs from four WT and four *Mfn2*^L643P/L643P^ mice at 16 weeks of age. The trabecular bone volume fraction was not significantly different between genotypes in either bone (Figure 5A). However, the trabecular bone thickness was significantly decreased in tibiae, but not femurs, of mutant mice compared to controls (Figure 5B). The thickness of the cortical bone was significantly decreased in both bones from homozygous mutants compared to controls (Figure 5C). Interestingly, while cortical bone area (Ct.Ar) and total area (Tt.Ar) were each unchanged between genotypes, the Ct.Ar/Tt.Ar ratio (cortical bone fraction) was significantly decreased in both the femur and tibia of mutant mice (Figure 5D–F) suggesting potential defects in bone remodeling. The changes observed in cortical and trabecular thickness suggest decreased osteoblast activity. Though cell-autonomous effects of MFN2 in bone have been described in the literature [35,36,54], the changes seen here could also be a result of defects in neuromuscular loading.

## 4. Discussion

An ENU-induced mutagenesis screen generated a mouse line carrying a recessive point mutation (T1928C) in exon 18 of the *Mfn2* gene, which results in a leucine to proline substitution (L643P). The mice were maintained by IVF due to poor breeding. Furthermore, while IVF efficiency was significantly reduced when using sperm from homozygous mutant mice, the genotypes of the progeny were of expected Mendelian ratios indicating a potential sperm defect, rather than a deleterious effect of the mutant allele on fetal viability. This observation also suggests that the L643P allele is not a complete loss of function, as homozygous *Mfn2* null mice die during gestation or shortly after birth [27,37,39].

Initial observations of homozygous mutants showed a phenotype with neuromuscular deficits, such as muscle atrophy, ataxia, sensory deficits, and decreased muscular endurance, indicating that these mice could potentially be a useful mouse model of CMT2A. However, further physiological and histological assessment of these mice pointed to disruption of muscle function, rather than the nerve dysfunction seen in CMT2A. Normal motor and sensory nerve conduction velocity, CMAP amplitude, the absence of muscle fiber type grouping or denervated NMJs, and a very small but statistically significant decrease in motor axon number suggest that these mice do not have a prominent peripheral neuropathy. However, the tibialis anterior muscles of homozygous mutant mice fatigued more quickly during repetitive stimulation and TEM of the same muscle showed decreased mean mitochondrial diameter, while significantly reduced MFN2 protein level and mtDNA content (a measure of mitochondrial health) were seen in medial and lateral gastrocnemius, respectively, of homozygous mutant mice. These results are consistent with disrupted MFN2 function in skeletal muscle, such as mitochondrial dynamics, maintaining mitochondrial genome integrity, membrane potential, respiration rates, and Ca^++^ homeostasis [17,55,56,57,58,59,60]. However, it is possible these mice also have more subtle defects in the cerebellum, spinal cord, or dorsal root ganglia that account for the sensory and gait defects observed.

MicroCT analysis of tibiae and femurs from these mice showed significant decreases in trabecular bone thickness of the tibia and cortical thickness and cortical bone fraction (Ct.Ar/Tt.Ar) of both the tibia and femur. This points to a potentially important role for *Mfn2* in bone development or maintenance, as others have reported increased expression of *Mfn2* during early osteogenesis [35,36,54]. Previous studies found that decreasing *Mfn2* expression in their respective precursor cells led to increased differentiation of osteoblasts and decreased differentiation of osteoclasts. Furthermore, with osteoblast precursor-specific *Mfn2*^KO^, cortical bone thickness *increased* compared to controls, a further indication that the L643P allele reported here is not a simple loss of function [36].

Despite reports of a similarly placed, recessive human mutation (R632W) that causes CMT2A [45], we did not find strong evidence of peripheral neuropathy in these mice, making them an unsatisfactory CMT2A model. However, as the first reported *Mfn2* mouse model with a pathogenic mutation in the TM domain, we believe that this mouse line will be useful to researchers studying MFN2 biology. The TM domain is critical for tethering MFN2 to the OMM, allowing the C-terminal HR2 domain to act as a redox sensor to induce mitofusin oligomerization within the OMM, while the N-terminal GTPase domain interacts with mitofusins on other mitochondria, allowing for the fusion of the OMM [13]. Therefore, the L643P mutation could affect mitochondrial fusion by disrupting MFN2 interactions in *cis* and in *trans*, or by causing aberrant localization. The introduction of proline in particular can cause problems with secondary structure due to its rigid, cyclic conformation. The diversity of phenotypes observed in these mice could also result from disruption of other cellular functions performed by MFN2 and highlights possible tissue-specific roles of this enigmatic enzyme.

## Figures and Tables

**Table 1:** List of protein coding genes located in the mapped interval, obtained using the Mouse Genome Informatics (MGI) Marker Query.

Chromosome	Start	End	cM	Strand GRCm39	MGI ID	Feature Type	Symbol	Name
4	148085179	148086531	78.66	+	MGI:97367	protein coding gene	*Nppa*	natriuretic peptide type A
4	148070264	148071662	78.57	+	MGI:97368	protein coding gene	*Nppb*	natriuretic peptide type B
4	149234448	149251162	78.96	−	MGI:97553	protein coding gene	*Pgd*	phosphogluconate dehydrogenase
4	149260776	149392150	79.05	−	MGI:108426	protein coding gene	*Kif1b*	kinesin family member 1B
4	149209491	149211220	78.89	−	MGI:109538	protein coding gene	*Cort*	cortistatin
4	149733625	149787023	80.15	−	MGI:1098211	protein coding gene	*Pik3cd*	phosphatidylinositol-4,5-bisphosphate 3-kinase catalytic subunit delta
4	149188603	149205104	78.87	+	MGI:1196227	protein coding gene	*Dffa*	DNA fragmentation factor, alpha subunit
4	148888886	149039346	78.87	+	MGI:1196251	protein coding gene	*Casz1*	castor zinc finger 1
4	149799832	149822501	80.15	−	MGI:1196277	protein coding gene	*Tmem201*	transmembrane protein 201
4	147945235	147953176	78.56	−	MGI:106506	protein coding gene	*Miip*	migration and invasion inhibitory protein
4	148123534	148144008	78.67	+	MGI:106639	protein coding gene	*Mthfr*	methylenetetrahydrofolate reductase
4	147953436	147954815	78.56	+	MGI:95595	protein coding gene	*Fv1*	Friend virus susceptibility 1
4	149552029	149569659	79.47	−	MGI:1913704	protein coding gene	*Nmnat1*	nicotinamide nucleotide adenylyltransferase 1
4	149602698	149650894	79.66	+	MGI:1915756	protein coding gene	*Ctnnbip1*	catenin beta interacting protein 1
4	149569686	149581125	79.53	+	MGI:1916401	protein coding gene	*Lzic*	leucine zipper and CTNNBIP1 domain containing
4	144144759	144165342	77.98	−	MGI:1916614	protein coding gene	*Cfap107*	cilia and flagella associated protein 107
4	148025352	148031771	78.57	+	MGI:1924284	protein coding gene	*2510039O18Rik*	RIKEN cDNA 2510039O18 gene
4	144118244	144135034	77.98	−	MGI:1924882	protein coding gene	*Pramel13*	PRAME like 13
4	148161518	148172488	78.67	−	MGI:1339977	protein coding gene	*Agtrap*	angiotensin II, type I receptor-associated protein
4	148088716	148123270	78.67	−	MGI:1347049	protein coding gene	*Clcn6*	chloride channel, voltage-sensitive 6
4	148696839	148711476	78.77	−	MGI:2387629	protein coding gene	*Tardbp*	TAR DNA binding protein
4	147958056	147989161	78.56	−	MGI:2442230	protein coding gene	*Mfn2*	mitofusin 2
4	148230173	148236592	78.67	−	MGI:1354743	protein coding gene	*Fbxo6*	F-box protein 6
4	148237256	148244663	78.67	−	MGI:1354744	protein coding gene	*Fbxo44*	F-box protein 44
4	148642886	148666858	78.76	+	MGI:1355322	protein coding gene	*Exosc10*	exosome component 10
4	149412873	149511206	79.08	−	MGI:1927086	protein coding gene	*Ube4b*	ubiquitination factor E4B
4	149534144	149539435	79.4	−	MGI:1890409	protein coding gene	*Rbp7*	retinol binding protein 7, cellular
4	148245078	148250881	78.68	+	MGI:2446216	protein coding gene	*Fbxo2*	F-box protein 2
4	144180341	144190326	77.98	−	MGI:2685281	protein coding gene	*Aadacl3*	arylacetamide deacetylase like 3
4	144396507	144412938	78.04	−	MGI:2685282	protein coding gene	*Aadacl4fm4*	AADACL4 family member 4
4	144503774	144513153	78.08	−	MGI:2685284	protein coding gene	*Aadacl4fm5*	AADACL4 family member 5
4	148727774	148756029	78.82	+	MGI:2685418	protein coding gene	*Gm572*	predicted gene 572
4	144246392	144255923	77.98	+	MGI:2685880	protein coding gene	*Aadacl4fm1*	AADACL4 family member 1
4	147757959	147787010	78.53	−	MGI:3650650	protein coding gene	*Zfp534*	zinc finger protein 534
4	144429761	144447974	78.05	−	MGI:3650721	protein coding gene	*AAdacl4fm3*	AADACL4 family member 3
4	145311770	145351915	78.22	+	MGI:3651014	protein coding gene	*Zfp268*	zinc finger protein 268
4	147056433	147075212	78.41	+	MGI:3651739	protein coding gene	*Zfp989*	zinc finger protein 989
4	147838431	147894245	78.54	−	MGI:3651978	protein coding gene	*Zfp984*	zinc finger protein 984
4	147390131	147418191	78.47	+	MGI:3651985	protein coding gene	*Zfp988*	zinc finger protein 988
4	147637734	147669655	78.51	+	MGI:3651986	protein coding gene	*Zfp985*	zinc finger protein 985
4	145237329	145265751	78.2	+	MGI:3652161	protein coding gene	*Zfp990*	zinc finger protein 990
4	144281570	144291704	77.99	−	MGI:3652194	protein coding gene	*Aadacl4fm2*	AADACL4 family member 2
4	144699192	144921575	78.17	−	MGI:2448530	protein coding gene	*Vps13d*	vacuolar protein sorting 13D
4	145595364	145626545	78.26	+	MGI:3649925	protein coding gene	*Zfp986*	zinc finger protein 986
4	144340277	144349968	78.01	+	MGI:3650257	protein coding gene	*Aadacl4*	arylacetamide deacetylase like 4
4	147907443	147932823	78.56	−	MGI:1922865	protein coding gene	*Zfp933*	zinc finger protein 933
4	148579737	148584919	78.76	−	MGI:3605801	protein coding gene	*Angptl7*	angiopoietin-like 7
4	146533487	146553897	78.31	+	MGI:3700963	protein coding gene	*Zfp992*	zinc finger protein 992
4	146586484	146623852	78.3	+	MGI:3700965	protein coding gene	*Zfp981*	zinc finger protein 981
4	147576874	147597943	78.5	+	MGI:3701121	protein coding gene	*Zfp982*	zinc finger protein 982
4	147445760	147475461	78.48	+	MGI:3701123	protein coding gene	*Zfp978*	zinc finger protein 978
4	149561883	149567796	79.51	+	MGI:3701129	protein coding gene	*Tmem274*	transmembrame protein 274
4	147216495	147265036	78.44	+	MGI:3701604	protein coding gene	*Zfp991*	zinc finger protein 991
4	146033882	146063194	78.37	+	MGI:3702694	protein coding gene	*Zfp987*	zinc finger protein 987
4	146093397	146135326	78.38	+	MGI:3705222	protein coding gene	*Zfp600*	zinc finger protein 600
4	145397267	145431007	78.23	+	MGI:3712454	protein coding gene	*Zfp980*	zinc finger protein 980
4	144099330	144104503	77.98	−	MGI:3712553	protein coding gene	*Pramel15*	PRAME like 15
4	146695418	146742617	78.28	+	MGI:3713585	protein coding gene	*Zfp993*	zinc finger protein 993
4	148518952	148529217	78.76	−	MGI:1918957	protein coding gene	*Ubiad1*	UbiA prenyltransferase domain containing 1
4	148214841	148230156	78.67	+	MGI:1919140	protein coding gene	*Mad2l2*	MAD2 mitotic arrest deficient-like 2
4	144993707	145041734	78.17	−	MGI:99908	protein coding gene	*Tnfrsf8*	tumor necrosis factor receptor superfamily, member 8
4	144940033	144973440	78.17	−	MGI:1314883	protein coding gene	*Tnfrsf1b*	tumor necrosis factor receptor superfamily, member 1b
4	144619397	144654779	78.14	+	MGI:1315215	protein coding gene	*Dhrs3*	dehydrogenase/reductase (SDR family) member 3
4	147106307	147145251	78.42	+	MGI:1328322	protein coding gene	*Rex2*	reduced expression 2
4	148687011	148699956	78.76	+	MGI:1330832	protein coding gene	*Masp2*	mannan-binding lectin serine peptidase 2
4	147994210	148021224	78.57	−	MGI:99907	protein coding gene	*Plod1*	procollagen-lysine, 2-oxoglutarate 5-dioxygenase 1
4	149212806	149222057	78.89	−	MGI:1917178	protein coding gene	*Cenps*	centromere protein S
4	148182894	148215155	78.67	−	MGI:1917683	protein coding gene	*Draxin*	dorsal inhibitory axon guidance protein
4	149828493	149858734	80.15	−	MGI:1917806	protein coding gene	*Slc25a33*	solute carrier family 25, member 33
4	148324721	148372422	78.73	−	MGI:2444403	protein coding gene	*Disp3*	dispatched RND transporter family member 3
4	149044992	149184300	78.87	−	MGI:1927868	protein coding gene	*Pex14*	peroxisomal biogenesis factor 14
4	148533068	148642140	78.76	+	MGI:1928394	protein coding gene	*Mtor*	mechanistic target of rapamycin kinase
4	149670925	149733356	79.91	+	MGI:1929895	protein coding gene	*Clstn1*	calsyntenin 1
4	148675970	148679076	78.76	+	MGI:102690	protein coding gene	*Srm*	spermidine synthase
4	149980740	150039463	80.46	−	MGI:1921896	protein coding gene	*Spsb1*	splA/ryanodine receptor domain and SOCS box containing 1
4	147696394	147726970		−	MGI:2148252	protein coding gene	*Zfp979*	zinc finger protein 979
4	146976154	146994032		−	MGI:5434766	protein coding gene	*Gm21411*	predicted gene, 21411
4	145130858	145178235		+	MGI:5625231	protein coding gene	*Gm42346*	predicted gene, 42346
4	146340962	146341306		−	MGI:6721541	protein coding gene	*Gm53232*	predicted gene, 53232
4	147303548	147311932		+	MGI:6723559	protein coding gene	*Gm54268*	predicted gene, 54268

**Table 2:** Decreased IVF efficiency using sperm from 13 affected male animals vs. WT(wild type) B6 males. B6 numbers for oocyte fertilization rate and number of pups produced are derived from ongoing IVF experiments at Novartis GNF at the time these data were recorded.

IVFs performed	13
Successful IVFs (pups produced)	5/13(38.4%)
Average oocyte fertilization rate	5.7% (vs. 65% for wild type B6)
Presumptive 2-cel embryos transferred	198
Number of pups produced	28 (14.1% vs. 50% for wild type B6)

**Table 3:** Tibialis anterior contractile properties for each genotype. N = WT (8F/7M), Het (9F/10M), Hom (10F/7M). Asterisks indicate significant difference from WT. * *p* < 0.05; ** *p* = 0.0001 by one-way ANOVA.

		Absolute Muscle Force			Normalized Muscle Force		
GenoType	Muscle Weight (mg)	Pt (g)	PO 50 Hz (g)	PO 150 Hz (g)	Pt (g/g)	PO 50 Hz (g/g)	PO 150 Hz (g/g)
WT	55.9 +/− 7.1	1.9 +/− 0.58	4.6 +/− 1.5	5.6 +/− 1.4	33.3 +/− 8	81.9 +/− 30.1	99.3 +/− 23.3
Het	56.5 +/− 10.2	1.9 +/− 0.66	4.4 +/− 1.7	5.3 +/− 1.8	34.2 +/− 12	79.1 +/− 29.8	96.4 +/− 29.8
Hom	** 42.9 +/− 6.8	* 1.4 +/− 0.48	3.4 +/− 1.3	* 3.8 +/− 1.4	33.1 +/− 11	88.1 +/− 30.2	89.4 +/− 31.1

## Data Availability

*Mfn2*^L643P^ mice are available with permission from GNF. Other reagents, and protocols are available upon request.

## Supplementary Materials

The following supporting information can be downloaded at: https://www.mdpi.com/article/10.3390/biology12070953/s1. Supplementary Movie. Affected male mutant mice and unaffected male littermates at 8, 12 and 16 weeks of age. Seconds 0–51, 8 weeks of age, the affected mouse starts in the back right and ends near the front left. The locomotor abnormality is subtle. Other mice are unaffected littermates (unknown mix of heterozygous and wild-type genotypes). Seconds 0:52–2:03, 12 weeks of age, the mouse on the right for the majority of the movie is the affected mouse. The other mouse is an unaffected littermate. The affected mouse moves much less and the locomotor defect is more obvious. Seconds 2:04-end, 16 weeks of age, the affected mouse starts in front and moves to the back left corner but moves very little compared to unaffected littermates.
